# CD4^+^ T-cell subsets in intestinal inflammation

**DOI:** 10.1111/imr.12039

**Published:** 2013-02-13

**Authors:** Matthew Shale, Chris Schiering, Fiona Powrie

**Affiliations:** 1 Sir William Dunn School of Pathology, University of OxfordOxford, UK; 2 Translational Gastroenterology Unit, Nuffield Department of Clinical Medicine, Experimental Medicine Division, John Radcliffe Hospital, University of OxfordOxford, UK

**Keywords:** T-cell differentiation, Treg, Th17, Th1, intestinal inflammation

## Abstract

Intestinal CD4^+^ T cells are essential mediators of immune homeostasis and inflammation. Multiple subsets of CD4^+^ T cells have been described in the intestine, which represents an important site for the generation and regulation of cells involved in immune responses both within and outside of the gastrointestinal tract. Recent advances have furthered our understanding of the biology of such cells in the intestine. Appreciation of the functional roles for effector and regulatory populations in health and disease has revealed potential translational targets for the treatment of intestinal diseases, including inflammatory bowel disease. Furthermore, the role of dietary and microbiota-derived factors in shaping the intestinal CD4^+^ T-cell compartment is becoming increasingly understood. Here, we review recent advances in understanding the multifaceted roles of CD4^+^ T cells in intestinal immunity.

This article is a part of a series of reviews covering CD4^+^ T-cell Subsets appearing in Volume 252 of *Immunological Reviews*.

## Introduction

The gastrointestinal tract is a major interface with the external environment and a site of high immune challenge. In addition to harboring a resident microbiota estimated to contain 10^14^ bacteria, as well as viral and fungal species, the intestine is continuously exposed to dietary and other ingested foreign antigens. On top of this, the gastrointestinal tract is also a key portal of entry for pathogens. To manage these diverse functions, it is populated by a complex and highly specialized network of innate and adaptive immune cells, including the largest population of T cells in the body.

The requirement to tolerate luminal contents while remaining poised to generate appropriate immune responses toward pathogens has led to the development of multiple layers of regulation 1, 2. Anatomical adaptations serve to physically limit the interaction of immune cells with luminal-derived antigens, whereas gut-specific conditioning factors regulate epithelial, stromal, and myeloid cells, generally favoring homeostasis. Multiple specialized lymphocyte populations are found within the intestinal tract, often demonstrating highly compartmentalized distribution and functional attributes. Among intestinal lymphocytes, CD4^+^ T cells represent a major population implicated in mediating diverse host protective and homeostatic responses. However, on the dark side, accumulation of CD4^+^ T cells in the intestine is also a key feature of inflammatory bowel disease (IBD) 3, 4. Through elaboration of cytokines that amplify and perpetuate inflammation, CD4^+^ T cells may be drivers of disease in IBD and as such represent an important therapeutic target.

In this review, we discuss recent advances in our understanding of the development, regulation, and function of intestinal CD4^+^ T cells, in both homeostatic settings and in disease. We particularly focus upon murine models of intestinal inflammation, and compare and contrast findings in these model systems with advances in understanding human disease. Finally, we discuss the translational and therapeutic implications of our growing knowledge of this important subset of cells.

## Intestinal T-cell subsets and their anatomical distribution

**Fig. 1:**
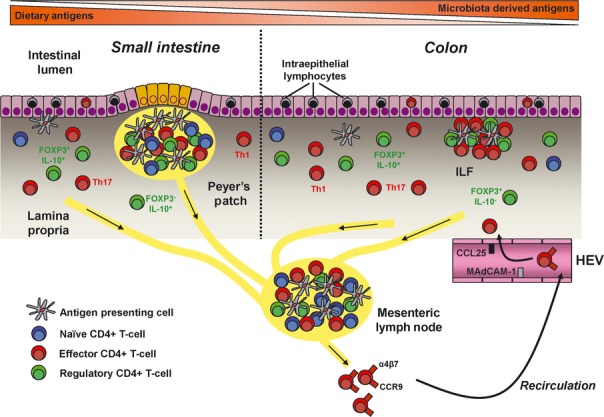
Anatomical distribution of intestinal CD4^+^ T-cell subsets At steady state, various populations of CD4^+^ T cells are distributed with the *lamina propria* of the intestine and associated inductive sites. The intestinal lumen contains are plethora dietary and microbiota-derived antigen, which are separated from the intestinal immune system by a single layer of epithelial cells. A specialized population of antigen-presenting cells within the intestine contributes to the generation of IL-10-producing regulatory T cells but also effector T cells expressing IL-17A or IFN-γ. Naive CD4^+^ T cells are abundant at inductive sites, but a small proportion of lamina propria CD4^+^ T cell also display surface markers associated with naive T cells. Trafficking of activated CD4^+^ T cells to the intestine is regulated by intestine-specific homing molecules. IL-10, interleukin-10; IFN-γ, interferon-γ; HEV, High endothelial venule.

Anatomically, CD4^+^ T cells are distributed within both immunological inductive and effector sites of the small and large intestine (*Fig.* 1). Inductive sites comprise the mesenteric lymph nodes (MLNs) and the gut-associated lymphoid tissue (GALT), including the Peyer's patches (PPs) of the small intestine, smaller isolated lymphoid follicles (ILFs) found throughout the intestines, as well as the cecal patch in mice, and the appendix in humans 5. The anatomy and function of organized lymphoid tissue in intestinal immunity has been reviewed in detail elsewhere 5–7.

In contrast, intestinal effector sites are characterized by the diffuse distribution of lymphocytes among non-immune cells and matrix, and include the intraepithelial (IEL) compartment and the *lamina propria* (LP). The composition of these effector sites demonstrates significant bias toward specific subsets of lymphocytes. Within the IEL compartment, the majority of T cells express CD8, either as the conventional CD8αβ heterodimer or as a CD8αα homodimer 8. Furthermore, the majority of such cells, at least within the small intestine, use a γδ T-cell receptor (TCR) rather than the conventional αβTCR. While CD4^+^ T cells, the majority of which express an αβTCR, are present within the IEL throughout the intestine, they comprise a greater proportion of T cells within more distal segments, including the colon 8, 9. Interestingly, IEL CD4^+^ T-cell populations show significant interstrain variation in mice that may reflect genetic or environmental control 9. Notably, infiltration of the IEL by CD4^+^ T cells is a feature of inflammation in experimental models of IBD.

Within the LP of both the small and large intestines, the majority of T cells are CD4^+^, with a smaller population of CD8αβ^+^ cells, although notably the human LP contains a greater proportion of CD8^+^ T cells compared with the murine gut 10, 11. Similar to their distribution within the IEL, CD4^+^ T cells may be more highly represented within the colonic LP. In addition to these conventional T-cell subsets, small populations of various unconventional cells, such as CD4^−^CD8^−^ T cells [including natural killer T (NKT) and mucosal-associated invariant T (MAIT) cells] are present in the healthy LP. The potential role of such cells in intestinal immunity and inflammation has been reviewed elsewhere 12, 13.

Within the steady-state LP of both the small intestine and colon, the majority of CD4^+^ T cells express a CD44^hi^CD62L^−^ effector memory phenotype of antigen-experienced cells 14, 15. Notable differences exist in the prevailing effector T-cell populations between anatomical niches within the intestine. Acquisition of distinct T-cell effector functions in intestinal niches is discussed in detail below. A small proportion of LP CD4^+^ T cells (up to 10% within the colonic LP) display surface markers associated with naive T cells 16. Whether these cells are tissue-resident or are undergoing normal trafficking through the LP is not fully defined, nor is whether they are able to undergo initial priming and differentiation within the LP. Indeed, the contribution of naive T cells in the LP to immunity in the intestine is an area worthy of further study.

## Intestinal T-cell homing

Myeloid antigen-presenting cells (APCs) of the intestine are a heterogeneous population consisting of dendritic cells (DCs) and macrophages. These populations are strategically positioned with the LP and in organized lymphoid structures and exhibit a number of adaptations associated with their dual role in tolerance and immunity in the intestine 17. DCs can act as a bridge with the adaptive immune system through their ability to acquire antigen in the intestine and migrate to the MLN where they prime the activation of naive CD4^+^ T cells 18. In addition to presenting antigen, intestine-derived DCs are specialized in their ability to prime T-cell responses that are focused on the intestine through the upregulation of gut-homing molecules on the responding T-cell surface 7. Expression of the ligands for these receptors on endothelial cells of postcapillary venules within the gut mediates intestinal homing. Specific receptors and their ligands demonstrated to be crucial for physiological T-cell homing in the intestine include the integrin α_4_β_7_ and mucosal addressin cell adhesion molecule-1 (MAdCAM-1), lymphocyte function-associated antigen-1, and intercellular adhesion molecule-1, very late antigen-4 (α_4_β_1_), and vascular cell adhesion protein 1, CCR9 and CCL25, and P-selectin glycoprotein ligand-1 and P-selectin 7, 19, 20. Notably, however, molecular aspects of T-cell homing have been largely studied in relation to the small intestine, and regulation of lymphocyte homing to the colon is less clear, particularly in the steady state. In the setting of inflammation, expression of α_4_β_7_ or α_4_β_1_ may contribute to the accumulation of T cells in the colon 20, 21. Similarly, the contribution of the CCR9/CCL25 axis in large intestinal homing and in accumulation within the small intestine in disease states requires further study.

## Intestinal homeostasis and consequences of its breakdown

Intestinal CD4^+^ T-cell populations can be broadly functionally divided into effector and regulatory populations. The lack of inflammation in the majority of individuals despite the enormous microbial and antigenic load within the intestine clearly demonstrates the dominance of regulatory mechanisms in the steady state 2. Similarly, the development of colitis in mice transferred with CD4^+^ T cells depleted of regulatory T cells confirms the functional importance of this subset in restraining cells which are otherwise capable of driving inflammatory disease 22–24.

When this balance is perturbed, inflammation results, as typified in humans by the IBDs, ulcerative colitis (UC), and Crohn's disease (CD) 25. These diseases, which affect around 1 in 1000 of the population in developed countries, are characterized by chronic inflammation of the intestinal tract, resulting in bloody diarrhea, abdominal pain, and weight loss. Although understanding of the etiopathogenesis of IBD has advanced rapidly in recent years, particularly through advances in genetics, treatment options remain limited and the majority of patients eventually require surgery. Furthermore, despite the development of increasingly efficacious therapies, such as monoclonal antibodies targeting tumor necrosis factor-α (TNF-α), such drugs have significant adverse event profiles, and are not curative 4.

Understanding of the underlying immunological basis of inflammation in IBD has benefited greatly from complimentary approaches using both animal model systems and studies of human tissue 4, 25. These studies demonstrate that regardless of the specific initiating factor, infiltration of the intestinal mucosa by CD4^+^ T cells is a critical step in the amplification and chronicity of disease.

## Intestinal effector CD4^+^ T cells

Early studies of the functional characteristics of murine LP CD4^+^ T cells described the presence of both interferon-γ (IFN-γ)^+^ T-helper 1 (Th1) and interleukin-4 (IL-4)^+^ Th2 cells, in the healthy and inflamed intestine. However, further studies in mice demonstrated that Th2 cells were largely absent from colonies free of parasitic infection 26. Rodent models of IBD dependent on either Th1 or Th2 responses have been described and characterized, and features aligning CD and UC to each subset have been highlighted 27. However, whereas IFN-γ^+^ Th1 cells could be clearly demonstrated in CD, IL-4^+^ cells were reported to be reduced in UC, although IL-5 and IL-13 were elevated in the inflamed intestine 28, 29. Later studies provided some explanation of these observations, with the finding of an increased population of IL-5 and IL-13 expressing non-classical CD1d-restricted NKT cells in active UC 29. More recently, description of the Th17 subset of CD4^+^ T cells has led to a reevaluation of the nature of the T-cell populations driving both experimental models of disease and human IBD. Characterized by expression of the transcription factor retinoic acid-related orphan receptor γt (RORγt) and the production of cytokines including IL-17A, IL-17F, IL-21 IL-22, granulocyte-macrophage colony-stimulating factor (GM-CSF), and in humans IL-26, Th17 cells have subsequently been shown to represent a major cellular population within both steady-state and inflamed intestinal mucosae 30.

## Intestinal Th1 cells

Critically required for adequate responses to intracellular bacteria and viruses, Th1 cells develop via a molecular program involving activation of signal transducer and activator of transcription 1 (STAT1) and STAT4 by IFN-γ and IL-12, respectively, which leads to the induction of T-bet, an essential transcription factor for the development of Th1 cells. T-bet drives the upregulation of the IL-12Rβ2 thereby increasing sensitivity to IL-12 and acts synergistically with STAT4 to drive optimal expression of IFN-γ 31, 32. The type I interferons IFN-α and IFN-β as well as the IL-12 family cytokine IL-27 can also contribute to Th1 differentiation through effects on T-bet induction 31, 33.

Murine models of chronic IBD, including colitis induced by the transfer of naive CD4^+^CD45RB^hi^ T cells into recombinase activating gene deficient (*Rag*^−/−^) hosts and bacterially driven intestinal inflammation in the context of IL-10 deficiency, can be effectively prevented by treatment with IFN-γ neutralizing antibody 34, 35. However, T-cell-derived IFN-γ appears to be dispensable for disease pathogenesis 36. Additional studies suggested a highly temporal dependency for IFN-γ in intestinal inflammation, with early but not late administration of an anti-IFN-γ antibody providing protection 34. A role for Th1 cells in intestinal immune pathology is further supported by findings that transfer of naive T cells deficient in key Th1-associated transcription factors T-bet or STAT4 fails to induce disease in the T-cell transfer model of colitis 36, 37. Together these data suggest either redundancy in the development and function of Th1 cells (i.e. STAT4 or T-bet-driven IFN-γ-independent responses) or that other subsets or mediators drive disease in their absence.

Accumulation of T-bet^+^ and IFN-γ^+^ CD4^+^ T cells is a feature of CD and nuclear extracts from mucosal T cells from patients with CD contain high levels of phosphorylated STAT4 and T-bet and increased expression of IL-12Rβ2 28, 37, 38. Interestingly, an anti-IFN-γ monoclonal antibody was shown to be ineffective in the treatment of established active CD, mirroring the observations from murine systems 39.

The role of the myeloid-derived cytokine IL-27 in regulating intestinal T-cell responses recently has received significant attention. In the steady state, IL-27-receptor deficiency is associated with reduced Th1 populations in the murine colon and MLNs 40. *In vitro*, IL-27 drives Th1 differentiation through STAT-1 and T-bet-dependent effects, and upregulation of IL-12Rβ2, implying that the IL-27/T-bet axis might be an important mediator of disease 41. Furthermore, single-nucleotide polymorphisms (SNPs) near the IL27 gene are associated with early onset IBD 42.

However, additional contrasting effects observed including suppression of effector T-cell proliferation, antagonism of Th2 and Th17 development, and augmentation of IL-10 production have been noted in other systems 43. In IL-10R knockout mice, deficiency of IL-27R significantly delayed but did not prevent the development of colitis and was associated with reduced expression of Th1-associated genes such as IFN-γ and IL-12p35 44. By contrast, in a T-cell transfer model of colitis, IL-27R^−/−^ T cells were impaired in their ability to induce intestinal inflammation, associated with reduced accumulation of IFN-γ^+^ Th1 cells within lymphoid tissues, including the MLNs 45. Intriguingly, Th1 populations in the colonic LP were largely unaffected. However, the most striking observation was the marked shift toward forkhead box protein 3 (Foxp3)^+^ T-regulatory cells (Tregs), in line with *in vitro* evidence that IL-27 can suppress TGF-β1-mediated Foxp3 induction in a cell-intrinsic STAT-3-dependent manner 46. The recent finding that IL-27 can also promote protective Treg responses in the intestine during infection with *Toxoplasma gondii* adds further complexity to the biology of this cytokine 47. Further understanding of the pleitropic effects of IL-27 in intestinal immunity and how this pathway may be manipulated therapeutically remains of significant interest.

## Intestinal Th17 cells

Studies of the role of the Th1-polarizing cytokine IL-12 in models of IBD demonstrated significant protection from disease to be afforded by antibody-mediated blockade of the IL-12p40 subunit 36, 48, 49. However, it was also noted that whereas IFN-γ blockade was only effective when administered at the onset of disease, treatment with anti-IL-12p40 could effectively treat established disease 48. Although initially interpreted as showing additional effects for IL-12 beyond driving IFN-γ production from Th1 cells, the description of IL-23, a novel heterodimeric cytokine comprising the IL-12p40 subunit combined with a novel IL-23p19 subunit, led to a re-evaluation of these findings 50. Studies using a variety of approaches to target the p19 subunit have now revealed IL-23 to instead be the critical cytokine required for the development of intestinal inflammation in a variety of both acute and chronic murine models 51–54. The functional receptor for IL-23 is composed of IL-12Rβ1 and the IL-23R subunit, and signals predominantly through Jak2-STAT3 but can also weakly activate STAT1, STAT4, and STAT5 55. It should be noted that limited information exists with regard to signaling events downstream of IL-23R and a detailed understanding of the IL-23 signaling pathway is currently lacking. Genome-wide association studies in human IBD have provided strong evidence of a functional role for the IL-23 axis in intestinal inflammation. SNPs linked to IBD have been identified in key components of this axis including *IL23R*, *IL12B* (encoding IL-12p40), *JAK2*, *STAT3*, and *CCR6* 56, 57.

Because Th17 cells have been implicated in the pathogenesis of IBD, it was originally thought that IL-23 is necessary for the generation of Th17 cells. However, it is now clear that IL-23 is dispensable for Th17 differentiation, and emerging data suggest that rather than driving Th17 development, IL-23 modulates Th17 effector function and pathogenicity 58–60. The development of Th17 cells in mice requires the combination of signals via the TCR and from TGF-β1, IL-1β, and a STAT-3-activating cytokine, such as IL-6 or IL-21^61^. Downstream of the TCR, the transcription factors BATF and IRF4 mediate chromatin accessibility, and together with STAT3, these factors initiate a transcriptional program that is modulated further through activation of the lineage-specific transcription factor RORγt, which drives expression of key genes in Th17 cell differentiation 62. Importantly, sustained expression of RORγt is required for the maintenance of the Th17 program as small molecule inhibitors of RORγt downregulate Th17 signatures genes, such as IL-17A and IL-23R, in established Th17 cells and inhibit Th17-driven immunopathology *in vivo*, making this pathway especially amenable to therapeutic intervention 63, 64.

In the steady state, Th17 cells preferentially locate to the LP of the small intestine and to a lesser extent the colon, where they produce IL-17A but not IFN-γ 65. In contrast to other mucosal sites, CD4^+^ T cells are the dominant IL-17A-expressing cells in homeostatic settings within the small intestine of adult mice 66. Interestingly, expansion of Th17 cell populations in the small intestine may occur in the setting of extraintestinal infectious or autoimmune disease, without detectable mucosal inflammation 67. Loss of Th17 cells into the intestinal lumen has recently been proposed as a mechanism controlling this population, although the general relevance of this remains to be established 67. The murine small intestine therefore appears to be an important site for the generation and regulation of Th17 cells. The basis for this observation might reflect the presence of specific antigens, distinct population of APCs, or the prevailing cytokine milieu, and is an intense area of study. The absence of Th17 cells in the intestines of germ-free mice provided an early indication of the critical role of components of the microbiota in the generation of such cells 66, 68, although conflicting reports as to the status of Th17 populations in the absence of intestinal bacteria have been published 69. The role of the microbiota in the development of Th17 cells is discussed in detail below.

The importance of the intestine as a site of Th17 cell development may reflect the prevailing cytokine environment, which is rich in TGF-β1, IL-1β, and IL-6. However, the persistent presence of large numbers of Th17 cells in the healthy intestine also argues against a simple, primarily pathogenic, function for Th17 cells. In the steady state, mice deficient in RORγt or STAT3 have markedly reduced Th17 cell numbers, although a small population persists, thought to develop via an alternative RORα-dependent pathway 65, 70–72. Studies of mice deficient in cytokines involved in Th17 differentiation indicate that important differences in developmental requirements may exist between intestinal and systemic populations. Based on studies in mice deficient in myeloid differentiation factor 88 (MyD88), which is downstream of Toll-like receptor (TLR) and IL-1R signaling pathways, initial reports concluded that Th17 cells were unaffected by the absence of IL-1β signaling 66, 68. In contrast, significantly attenuated numbers of intestinal Th17 cells were seen in mice deficient in IL-6, despite normal peripheral populations 65, 73. By contrast, it was recently reported that IL-6 may be dispensable for the development of steady-state intestinal Th17 cells, but that IL-1β is both necessary and sufficient to drive mucosal Th17 accumulation in a cell-intrinsic manner, but has less consistent effects on systemic populations 74. These seemingly contrasting findings may reflect differences in the definition of Th17 cells. The latter study used a reporter-mouse approach and defined Th17 cells based on expression of Rorγt^gfp^, whereas earlier studies used IL-17A production following *ex vivo* restimulation to quantify Th17 cells, an approach that may overestimate the prevalence of such cells 75. Intestinal Th17 cell numbers are maintained or even expanded in IL-21R-deficient mice, confirming results in inflammatory settings where the contribution of IL-21 to Th17 differentiation is only revealed in the absence of IL-6 61, 66. The intestines of mice deficient in TGF-β1, or having impaired ability to activate the latent form of TGF-β1 have been reported to be effectively devoid of Th17 cells 66, 76. However, more recently it has been reported that murine Th17 cells may not require TGF-β1 for their development 59.

The factors controlling intestinal Th17 accumulation in states of inflammation may differ from those operating in the steady state. For example, IL-1R-deficient CD4^+^ T cells are unaffected in their ability to differentiate to Th17 cells, but are impaired in their accumulation in the colonic LP in T-cell transfer colitis 77. In addition, in a model of chronic inflammation of the central nervous system-specific deletion of TGF-β1 in T-effector cells was shown to prevent the development of disease, suggesting that Th17 cells may amplify their own differentiation in an autocrine manner 78. Thus, the *in vivo* cytokine requirements for Th17 development in the intestine and beyond require further clarification.

The lack of clarity around this issue may reflect the functional heterogeneity of Th17 cells studied in different anatomical locations and in homeostasis and inflammation. Historically, Th17 cells have been defined by production of IL-17A; however, it is increasingly apparent that the functional characteristics of these cells are determined by the combination of a number of cytokines. Studies of *in vitro*-differentiated cells, and those generated in mice in the absence of specific cytokines involved in Th17 development, suggest significant differences may exist in the pathogenic potential of the resulting T cells 58, 79. Examination of the transcriptional profile of such cells has revealed signatures associated with pathogenicity in models of extraintestinal inflammatory disease 59. A recent study by Kuchroo *et al*. 80 identified TGF-β3 as a novel target of IL-23 in CD4^+^ T cells. The authors found that TGF-β3, mainly produced by Th17 cells themselves in response to IL-23, drives a distinct transcriptional signature in Th17 cells that is associated with heightened pathogenic potential. Although the role of TGF-β3 in intestinal inflammation is currently unclear, the important question of the relationship of intestinal steady-state Th17 cells to those implicated in driving intestinal inflammation clearly requires further study, including definition of their corresponding transcriptional and functional attributes.

In both mice and humans, impaired Th17 responses through deficiency in critical molecular components of this axis are associated with disease. Mutations in *STAT3* result in hyper-immunoglobulin E (IgE) syndrome (Job's syndrome), characterized by a marked reduction in the number of IL-17-secreting cells in the periphery and susceptibility to recurrent *Staphylococcal* and fungal infection 81, 82. Impaired Th17 development due to gain-of-function *STAT1* mutations is associated with chronic mucocutaneous candidiasis 83, a disease also seen in patients with circulating neutralizing antibodies against key effector cytokines of the Th17 axis, including IL-17A, IL-17F, and IL-22 84. Together, these data indicate that Th17 cells are an important component of host defense at mucosal surfaces including the intestine.

However, accumulation of IL-17A-expressing cells in the mucosa is an important feature of numerous models of intestinal inflammation. In human CD, Th17-associated molecules such as IL-17A, IL-17F, IL-21, IL-22, CCR6, and IL-23R are increased compared with controls or UC patients; however, similar elevations in UC have been noted by other groups 85.

## Mediators of Th17 effector T-cell function

Although a role for the IL-23/Th17 pathway has been convincingly demonstrated in multiple murine models of intestinal inflammation, the roles of its downstream mediators are less well understood. Whereas the transfer of wildtype (WT) naive CD4^+^ T cells into *Rag*^−/−^ hosts results in intestinal inflammation and wasting, the transfer of RORγ^−/−^ or IL-23R^−/−^ T cells, or the use of IL-23p19^−/−^ recipient mice, results in highly attenuated intestinal disease 60, 86, 87. In mice receiving WT T cells, significant elevation in multiple Th17 cytokines is observed in the intestinal mucosa. By allowing the transfer of T cells deficient in individual cytokines, this model is a particularly elegant system in which the specific role of CD4^+^ T-cell-derived cytokines in intestinal inflammation can be examined.

## IL-17A and IL-17F

Using this approach, multiple groups have now demonstrated that T cells deficient in IL-17A are unimpaired in their ability to drive both the intestinal and systemic features of this model 86–88. Indeed, transfer of IL-17A-deficient T cells may result in an exacerbated wasting phenotype, proposed to occur through a T-cell-intrinsic requirement for IL-17A signals to suppress IFN-γ production 89. In contrast, disease induced by Treg-specific STAT3 deficiency was significantly attenuated by anti-IL-17A treatment indicating a pathogenic role in some settings 90. Similar mixed findings have been observed using chemically induced models of colitis where epithelial injury is a prominent feature of the pathogenesis. Thus, in dextran-sodium sulfate (DSS) colitis, both protective 91, 92 and pathogenic roles 93 for IL-17A have been documented. However, chemically driven models of disease are T-cell independent, somewhat complicating the interpretation of these findings.

A number of the functions of IL-17A, such as prominent effects on neutrophil recruitment, are shared by the related cytokine IL-17F, as both cytokines utilize a common heterodimeric signaling receptor composed of IL-17RA and IL-17RC subunits 94. Studies in T-cell transfer colitis suggest redundant effects of these cytokines in driving colitis as only concomitant ablation of IL-17A and IL-17F resulted in attenuated colitis 86. In *Citrobacter rodentium* infection, a murine model of enteropathogenic *Escherichia coli* infection, both IL-17A and IL-17F exert protective effects 95. However, in other systems, such as DSS colitis, the function of IL-17F appears to oppose that of IL-17A, exacerbating disease 92. Together the data paint a complicated picture of the roles of IL-17A and IL-17F in intestinal inflammation that encompasses both redundancy and a contextual nature that may be dictated by specifics of the model of inflammation or environmental conditions.

Interestingly, recent experience in a clinical trial of an anti-IL-17A monoclonal antibody in active CD appears to confirm a similar redundancy or possibly even protective effect for IL-17A in established human disease 96.

## IL-22

IL-22 is produced by Th17 cells as well as other T cells and appears to have an IL-23 dependency 97. In IL-23p19^−/−^ mice, whereas overall IL-17A^+^ CD4^+^ T cells are relatively unaffected, a specific reduction in IL-17^+^IL-22^+^ cells has been reported 66. In human IBD, serum IL-22 levels are increased in active disease, and may correlate with disease-associated *IL23R* polymorphisms 98, 99. Interestingly, it has been reported that remission in UC is associated with the presence of IL-17A^+^IL-22^+^CD4^+^ T cells, in contrast to the absence of IL-22-producing cells in active disease 100.

IL-22R is highly expressed on non-hematopoietic cells of the intestine, including stromal and epithelial cells, and activates STAT3 to promote the elaboration of antimicrobial peptides, including RegIIIβ, RegIIIγ, and mucins 97. IL-22 has been reported to play a protective role in experimental models of IBD, including CD45RB^hi^ T-cell transfer, DSS colitis, and in the spontaneous intestinal inflammation developing in TCRα^−/−^ mice 101, 102. Importantly, whereas IL-22 is protective in the *Citrobacter rodentium* model of acute colitis 101, non-Th17 cell sources may be critical, including CD4^+^ lymphoid tissue-inducer (LTi) cells 103. In contrast with these protective properties, IL-22 has been shown to promote colitis in some settings. Colitis induced in *Rag*^−/−^ mice transferred with Treg-depleted CD45RB^lo^ memory T cells is in part driven by IL-22 104. Similarly, colitis induced by *Helicobacter hepaticus* infection is promoted by IL-22 in both innate and lymphocyte replete models (authors' unpublished observations). In reconciling these apparently contradictory results, it is interesting to note that highly context-specific effects of IL-22 are seen in experimental lung inflammation, dependent upon the presence of IL-17A 105. Such context-specific effects await exploration in the setting of intestinal disease.

## IL-21

The role of IL-21 in intestinal inflammation is less well characterized. IL-21 is both an important differentiation factor and a product of Th17 cells, and it may prevent Treg development 61. In both acute and chronic DSS-induced disease and in colitis induced by treatment with trinitrobenzene sulfonic acid (TNBS), IL-21 has been shown to be pathogenic 106, 107. Mechanisms for this may include effects on the generation of Th17 cell responses and upon intestinal chemokine secretion and metalloproteinase production. However, while IL-21 is an important Th17 cell mediator, it is produced in large quantities by other cells, including NK cells 61. In the intestine in active human IBD, the majority of IL-21-producing cells are IFN-γ^+^ Th1 cells and are not IL-17A^+^ 108. Therefore, any specific requirement for Th17 cell-derived IL-21 in disease pathogenesis remains to be further defined.

## GM-CSF

GM-CSF has recently been proposed to be a critical pathogenic effector of Th17 responses in experimental autoimmune encephalomyelitis (EAE), a murine model of multiple sclerosis 109, 110. In this model, GM-CSF is produced by both Th1 and Th17 cells, with production downstream of IL-1β and IL-23. In the context of intestinal inflammation, antibody-mediated neutralization of GM-CSF ameliorates disease 111, whereas GM-CSF-deficient T cells were unimpaired in their ability to induce colitis 77, suggesting that T cells may not be an essential source of GM-CSF in the intestine. The colitogenic potential of GM-CSF involves the accumulation of granulocyte-macrophage progenitors in the intestine linking an inflammatory Th17 response to dysregulated hematopoiesis 111.

## Plasticity of intestinal effector T cells

It is increasingly clear that Th17 cells present in the intestine during inflammation may differ from steady-state populations in terms of their effector functions. Whereas steady-state Th17 cells are largely IL-17A single positive, significant proportions of the cells accumulating in inflammatory settings produce additional mediators such as IFN-γ or GM-CSF 60, 109, 110. Human Th17 cells display differential cytokine secretion patterns depending on the eliciting pathogen. Whereas Th17 cells generated in response to *Candida albicans* produce IL-17A and IFN-γ, *Staphylococcus aureus*-specific Th17 cells produce IL-17A and the immunoregulatory cytokine IL-10 112. In mice, colitis is associated with the accumulation of IL-17A^+^IFN-γ^+^ CD4^+^ T cells in the mucosa in an IL-23-dependent manner 60. In this scenario, IL-23 functions through effects on proliferation within the intestine, as well as directly promoting the emergence of IL-17A^+^IFN-γ^+^ cells in a cell-intrinsic manner. Interestingly, abrogation of IL-23 signaling does not affect the presence of IL-17A^+^IFN-γ^−^ or IL-17A^−^IFN-γ^+^ cells in this model.

IL-23 signals into Th17 cells were shown to drive IFN-γ production in a STAT4 and T-bet-dependent manner *in vitro* 113. Therefore, an important question to ask is what relationship mucosal IFN-γ single-positive cells have to Th17 cells in terms of *in vivo* plasticity, as the developmental pathways of Th1 cells present in the steady-state or inflamed intestine are incompletely defined. Recently, fate mapping experiments of IL-17A-producing T cells during the course of EAE demonstrated that the majority of IL-17A^+^IFN-γ^+^ and IFN-γ single-producing cells arise from Th17 cell progeny 75. Importantly, this transition of Th17 cells into IFN-γ-producing T cells required IL-23 and correlated with increased expression of T-bet. A similar experimental approach will be required to delineate the origins of IFN-γ^+^ CD4^+^ T cells in homeostatic and inflammatory settings in the intestine and could provide important mechanistic insights into the role of plasticity in intestinal immunity. Significantly, the emergence of a population of T cells capable of producing both IL-17A and IFN-γ has also been described in intestinal biopsies of IBD patients 114, 115.

Human T-cell clones derived from the mucosa of patients with CD were able to convert from Th17 or Th1/Th17 phenotypes to IL-17A^–^IFN-γ^+^ Th1 cells following *in vitro* stimulation with IL-12, indicating similar plasticity or progression may occur in human T cells, although the specific signals required may differ 114. In this regard, murine studies have highlighted the importance of context and prevailing cytokine environment in regulating Th17 to Th1 transition. Stimulation of Th17 cells with IL-12 could drive the acquisition of a Th1 phenotype *in vitro* even in the presence of TGF-β1; however, IL-23 could exert a similar effect only in the absence of TGF-β1 113. Furthermore, continued antigenic stimulation via the TCR also favored progression from a Th17 to Th1 phenotype *in vitro*. Such studies highlight the important role of TGF-β1 in controlling T-cell fate.

The emergence of mixed Th1/Th17 cells in the inflamed mucosa, in contrast to their absence in the steady state suggests such cells may contribute to the pathogenesis of disease. Interestingly, in disease induced by the transfer of *CBir*-specific T cells, recognizing bacterial flagellin, severity correlated with the proportion of Th1/Th17 cells present in the LP. Furthermore, using *in vitro*-differentiated cells polarized to Th1 or Th17 phenotypes, it was shown that whereas transfer of Th1 cells resulted in mild disease, transfer of similar numbers of Th17 cells induced severe colitis 116, 117. Analysis of the colons of animals receiving Th17 cells revealed populations of Th17, Th1/Th17, and Th1 cells, demonstrating the potential for plasticity of Th17 cells in the intestine *in vivo*. Although T-cell plasticity has been studied extensively *in vitro,* the anatomical sites relevant to intestinal T-cell plasticity remain unknown.

In addition to the ability to adopt a Th1/Th17 phenotype, Th17 cells may also acquire IL-10 production under appropriate circumstances, a phenomenon reported for all known lineages of effector T cells and thought to be a feature of continued antigenic stimulation 118. Acquisition of IL-10 production by Th17 cells may be an important regulatory mechanism for such cells, as suggested by the appearance in the intestine of IL-17A^+^IL-10^+^ T cells following treatment with anti-CD3 antibody, which is known to result in an immunosuppressive environment within the intestinal mucosa 67. While further definition of the role of such cells in intestinal immunity is awaited, in experimental neurological inflammation exposure of Th17 cells to IL-23 limits IL-10 production and promotes pathogenicity of Th17 cells, suggesting that inhibition of IL-10 might abrogate the regulatory capacity of Th17 cells 79. Beyond an ability to express IL-10, Th17 cells exhibit a specific developmental relationship with Foxp3^+^ inducible Tregs (iTregs), discussed in detail below.

## Intestinal regulatory CD4^+^ T cells

Despite the presence of a large population of potentially pathogenic effector T cells in close proximity to the largest antigen load in the body, the majority of individuals do not develop intestinal inflammation. This clearly points to the presence of dominant suppressive and regulatory mechanisms, restraining innate and T-cell responses in the steady state 2. In mice, early adoptive transfer experiments demonstrated such regulatory activity to be a function of CD4^+^ T cells contained within the CD45RB^lo^ population of antigen-experienced cells, in particular those expressing CD25, the α-chain of the IL-2 receptor 119. Cotransfer of CD45RB^lo^ or CD25^+^ cells prevented the development of colitis normally seen with the transfer of naive T cells alone and could cure disease in some settings. These observations established the concept of an important functional role for CD4^+^ Treg cells in maintaining intestinal homeostasis and led to efforts to identify further phenotypic markers and effector mechanisms utilized by regulatory populations. A number of subsets of T cells possessing regulatory or suppressive activity have now been characterized, but those expressing the transcription factor Foxp3 and IL-10-producing cells appear to be of particular functional importance in intestinal homeostasis and the control of inflammation 2.

In comparison with systemic immune compartments, the intestine is enriched for the presence of Treg cells. In the colon of specific pathogen-free (SPF) mice, approximately 30–40% of CD4^+^ T cells produce IL-10, with the overwhelming majority coexpressing Foxp3 120. Although IL-10^+^Foxp3^+^ Treg cells are also found in abundance in the small intestinal LP, a sizable fraction of IL-10^+^ CD4^+^ T cells in this location do not express Foxp3, exhibiting a Tr1 phenotype 121. Notably, such cells are also the major IL-10-producing population within the small intestinal IELs, but are rare at any site within the colon. These data support the concept of highly compartmentalized regulatory cell populations and suggest overlapping, but non-redundant functions for these cells.

The anatomical basis of the interaction between regulatory and effector T cells is incompletely understood. In the prevention model of T-cell transfer disease, in which Treg cells are cotransferred with naive CD45RB^hi^ CD4^+^ cells, both cell types preferentially accumulate in lymphoid tissues outside of the intestine 122. By contrast, where Treg cells are transferred into mice with established disease, regulation appears to occur within the intestinal LP 120, 123. Precisely how specific anatomical interactions between effector and regulatory populations influence regulatory T-cell adaptation and function is an important area for further study.

## Foxp3^+^ Treg cells

An essential functional role for Foxp3 in restraining inflammatory responses in the normal intestine is demonstrated by the development of intestinal inflammation as a key feature of disease in the immune dysregulation, polyendocrinopathy, enteropathy, X-linked syndrome, which develops in humans with germ line mutations in Foxp3 124. Natural Treg cells (nTregs) develop in the thymus under the influence of appropriate TCR stimulation, which activates NF-κB-dependent pathways and suppresses the phosphoinositol 3-kinase-Akt-mammalian target of rapamycin pathway. Further development requires signals from the costimulatory molecule CD28 and common γ chain cytokines, including IL-2 and IL-15, to activate STAT5 125. Expression of Foxp3 is considered essential to the regulatory properties of these cells.

In addition to thymus-dependent nTreg cells, the intestinal Foxp3^+^ cell population contains a large population of non-thymically derived Foxp3^+^ iTreg cells. Although nTreg and iTreg cells have been shown to have different transcriptional signatures 126, 127, they are hard to distinguish once generated. Recently, markers including Helios, a member of the Ikaros family of transcription factors, and the transmembrane protein neuropilin-1 have been reported to permit segregation of iTreg from nTreg cells 128–130. Although the validity of such markers continues to be debated, it is notable that up to 45% of total colonic Foxp3^+^ cells in the intestine can be defined as iTregs by such criteria. Such cells are conspicuously reduced in the colons but not MLNs of germ-free mice, and reconstitution with components of the normal microbiota drives the accumulation of Foxp3^+^ cells with a Helios^–^Neuropilin-1^–^ iTreg phenotype in the colon 129.

Recent studies have given some insight into the relative contribution of nTreg and iTreg cells to homeostasis. Specific deletion of the conserved non-coding sequence CNS1 in the Foxp3 locus demonstrated that this element was specifically required for iTreg induction, whereas its deletion did not affect nTreg development 131. Surprisingly, loss of Foxp3 CNS1 led to exacerbated Th2-type immunopathology in the lung and intestine, demonstrating that iTreg cells are particularly important for restraining Th2-type responses at mucosal sites 132. In addition, cooperation between iTreg and nTreg cells appears to be required for protection from the autoimmune disease that develops in Foxp3-deficient mice 133, and protection from colitis induced by transfer of naive CD4^+^ T cells 127. However, the molecular basis of this cooperation is currently unknown.

The large iTreg population in the intestine might reflect the presence of specialized populations of APCs favoring iTreg development 18. Alternatively, this might also reflect the presence of their cognate antigen at that site, whether host or exogenous in nature. Studies of TCR specificities among CD4^+^ T cells recovered from steady-state mice reveal that little overlap exists between regulatory and effector populations 134. Furthermore, TCR repertoires expressed by intestinal Treg cells may differ significantly from cells isolated from secondary lymphoid tissues, with a significant proportion of the most highly represented Treg-TCRs in the colon reported to show marked reactivity against components of the microbiota 135. Interestingly, in the setting of colitis, some CD4^+^ effector T cells express TCRs normally associated with steady-state Treg cells, suggesting that in the appropriate inflammatory environment, such cells might be skewed toward a highly pathogenic effector phenotype.

## IL-10^+^ Treg cells

Among multiple regulatory mechanisms described for Foxp3^+^ Treg cells, production of the immunosuppressive cytokine IL-10 is a key pathway. Whereas in the colon Foxp3^+^ cells are the almost exclusive T-cell source of IL-10, in the small intestine Foxp3-IL-10^+^ cells (Tr1 cells) may play an important role 136. Furthermore, IL-10 production by cells primarily demonstrating an effector phenotype is well recognized. IL-10-producing Th1, Th2, and Th17 cells have all been described, with their generation apparently related to chronic immune stimulation 118. However, many IL-10-producing cells, particularly within the small intestine, do not produce additional effector cytokines, and express a more Tr1-like phenotype. Whether this is related to a particular state of differentiation or a *bona fide* functionally specialized T-cell subset remains to be established.

The contrasting phenotypes seen in Foxp3 or IL-10 deficiency point to non-redundant functions for each molecule and cell type in aspects of intestinal immune regulation. Whereas Foxp3-deficient mice develop fatal autoimmunity, ablation of the IL-10 gene renders mice susceptible to intestinal inflammation driven by otherwise non-pathogenic components of the intestinal microbiota, such as *Helicobacter hepaticus* 136. Notably, intestinal disease in IL-10^−/−^ mice is dependent on the presence of CD4^+^ T cells and does not occur in Rag^−/−^ or CD4-depleted animals 137.

The cellular targets of IL-10 in suppressing intestinal inflammation are increasingly understood. Suppression of Th1 CD4^+^ T cells appears not to occur in a cell-intrinsic fashion, which corresponds with the description of low levels of IL-10R expression on such cells 138, 139. Instead effects on APCs, including downregulation of activation, major histocompatibility complex class II antigen presentation, and the expression of costimulatory molecules have been proposed to underlie suppression of Th1-dependent responses 140. However, recent studies suggest this mechanism may be of less importance in Th17-driven disease. High levels of expression of IL-10R on this subset compared with other effector T cells points to a potential direct role for IL-10 signaling in controlling Th17 responses 139.

Using mice expressing a dominant negative-IL-10Ra subunit under control of the CD4 promoter (resulting in T-cell-specific ablation of IL-10 signaling), it was shown that the emergence of intestinal IL-17A^+^ and IL-17A^+^IFN-γ^+^ cells following anti-CD3 treatment was directly inhibited by cell-intrinsic IL-10 signals. In contrast, IFN-γ^+^ Th1 cells were similar in number to those seen in WT mice 139. In models of colitis driven by IL-17A^+^ or IL-17A^+^IFN-γ^+^ T cells, suppression of disease by Foxp3^+^ Treg or Tr1 cells required direct IL-10 signaling into the effector T cells. Interestingly, Foxp3^+^ cells lacking a functional IL-10R are unable to suppress Th17 cell-driven colitis 141. Similar observations have been made using Treg cells lacking STAT3, a mediator of both IL-10 signaling and IL-10 production in Tregs 90. These results suggest that in addition to direct activity on Th17 effector cells, Treg cells are themselves important targets of IL-10 signaling in the *in vivo* control of inflammatory responses.

The key role of IL-10 in intestinal homeostasis revealed in mouse models has also been validated in human IBD. Loss of function mutations in *IL10*, *IL10RA*, or *IL10RB* is responsible for severe early onset IBD in some infants 142. Furthermore, SNPs in *IL10* have been associated with adult IBD in GWAS studies^25^.

## TGF-β1

TGF-β1 plays a critical role in the development and function of Treg cells, including Foxp3^+^ and Tr1 cells 2. Despite the abundance of other cellular sources within the intestine, T cells may represent a non-redundant population of TGF-β1-producing cells, as CD4^+^ T-cell-specific TGF-β1 deficiency can result in spontaneous colitis 143, 144. However, in T-cell transfer models, TGF-β1^−/−^ Treg cells may retain the ability to suppress disease, although conflicting reports exist in this regard 136. Naive T cells that are unable to respond to TGF-β due to expression of a dominant negative (DN) TGF-βII receptor are refractory to control by Treg cells and induce severe intestinal inflammation 145. Similarly, overexpression of Smad7, which inhibits TGF-β1 signaling, results in colitogenic T cells resistance to suppression by Treg cells 146. Notably, T cells from patients with IBD exhibit upregulation of SMAD7. Together the data highlight the role of sensitivity of target T cells to TGF-β for appropriate Treg-mediated control raising the possibility that interference with the SMAD7 pathway may be a therapeutic target in IBD 147.

## IL-35

IL-35 is a recently described heterodimer cytokine expressed at high levels by CD4^+^ Foxp3^+^ Treg cells 148, 149. Comprised of the IL-12p35 subunit of IL-12 and Epstein–Barr virus-induced gene-3 (Ebi-3), itself a component of IL-27, IL-35 is produced at high levels by activated Treg cells during suppression of effector T cells *in vitro*. Treg cells rendered deficient in IL-35 activity by genetic deletion of *Il12a* (encoding IL-12p35) or *Ebi3* are impaired in their ability to cure intestinal and systemic features of disease in the CD45RB^hi^ T-cell transfer model 149. Furthermore, IL-35 signaling into conventional T cells may induce a suppressive Foxp3^–^ phenotype characterized by production of IL-35 but not IL-10 or TGF-β, which also demonstrates suppressive activity in the cure model of T-cell transfer disease 150. The role of IL-35 in human Treg function is less well documented, although production of biologically active IL-35 from human T cells has been reported 150, 151.

## OX40

OX40 is a member of the TNF superfamily of costimulatory molecules, whose expression was originally described on activated conventional T cells, where it may have a functional role in T-cell memory responses 152. However, OX40 expression has also been noted on Treg cells, particularly among colonic Foxp3^+^ populations 153. Whereas OX40 deficiency is associated with reduced steady-state colonic Treg cell populations, it appears to be redundant for their accumulation in inflammatory settings. However, OX40^−/−^ Tregs are unable to suppress disease when cotransferred with CD45RB^hi^ naive T cells, implying a functional requirement for OX40 to control inflammatory effector pathways. Notably, OX40 also promotes colitogenic Th1 responses, highlighting the overlapping costimulatory requirements of effector and regulatory responses 153.

## Intestinal regulatory and effector T-cell relationships

Cells coexpressing RORγt and Foxp3 are found in intestinal tissues 154, 155. Notably, both Treg cells and Th17 cells require TGF-β1 for their development, and the presence or absence of further factors, including the STAT3-activating cytokines IL-6 and IL-23, may determine the balance of these populations in the steady-state or inflamed intestine 156. IL-23 favors the accumulation of Th17 cells in the inflamed intestine at the expense of Foxp3^+^ Treg cells in a cell-intrinsic manner 60. Similarly high levels of IL-6, as seen in inflammation, favor a Th17 response, and anti-IL-6R treatment increases the proportion of CD4^+^ T cells expressing Foxp3 in intestinal and peripheral tissues 61. Foxp3^+^ Tregs have also been reported to express key transcription factors associated with other effector T-cell subsets, including STAT3, T-bet, IRF-4, and GATA3 90, 157–159. Expression of these transcription factors by Foxp3^+^ Treg cells appears to be an essential component of their adaptation to particular environmental conditions and important for their ability to effectively control specific types of immune responses.

The acquisition of transcription factors more normally associated with effector T cells raises the question of the stability of the immune-suppressive Foxp3-driven program. This is currently hotly debated, and although Foxp3^+^ cells have been shown to acquire effector functions 160, the physiological importance of this in intestinal inflammation remains to be established.

## Regulation of the development of intestinal CD4^+^ T-cell subsets

**Fig. 2:**
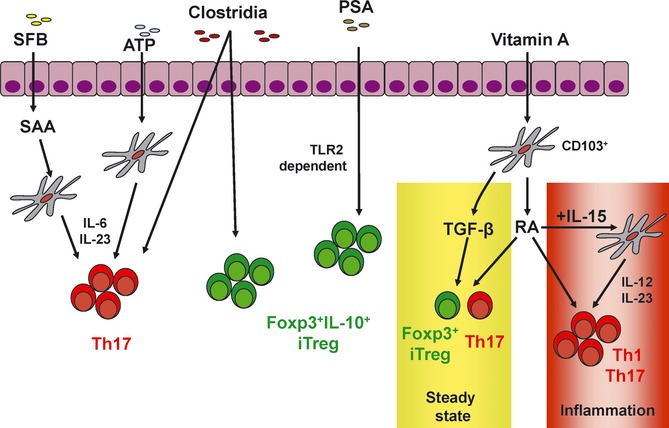
Regulation of intestinal CD4^+^ T-cell subsets by environmental factors The microbiota directs the accumulation of both Treg cells and Th17 cells in the intestinal *lamina propria*. Clostridia species induce IL-10-producing iTregs. Colonization with the human commensal Bacteroides fragilis leads to induction of iTregs and IL-10 production through PSA–TLR2 interactions. Segmented-filamentous bacteria promote the induction of Th17 responses, possibly by promoting expression of IL-6 and IL-23 from antigen-presenting cells through serum amyloid A. The vitamin A metabolite retinoic acid can promote both iTreg and effector T-cell differentiation, depending on the prevailing cytokine environment. The relative balance of Treg and Th17 cells can alter the outcome of local as well as systemic immune responses. IL-10, interleukin; PSA, polysaccharide A; TLR, Toll-like receptor.

The intestine is a site of immense immune challenge, home to a dense microbiota, numbering up to 10^14^ bacteria, as well as viral and fungal components, and a vast array of dietary-derived antigens, adjuvants, and metabolically active components. Unsurprisingly, it has long been recognized that environmental factors influence intestinal T-cell populations and their responses (*Fig.* 2). Prime among such environmental factors is commensal bacteria. In addition to other immunological defects, germ-free mice exhibit failure of development of GALT and reduction in LP T-cell populations 161. Conventionalization of such animals, by cohousing with specific pathogen-free mice, results in development of normal LP T-cell numbers. However, knowledge of the contribution of individual constituents to this process is limited. Recent evidence suggests that species-specific differences exist in the requirement for specific components of the microbiota for immune maturation. However, the mechanisms underlying this are poorly understood.

## Th17 cells

Whereas defects in all intestinal T-cell subsets are seen in germ-free mice, a specific deficit in Th17 was noted early in the study of factors involved in their development 66, 68. Using germ-free colonies and mice obtained from multiple commercial sources, it was noted that Th17 cells were absent from the intestines of both germ-free mice and specific pathogen-free animals obtained from Jackson Labs 66. Cohousing Jackson Labs mice with those from other commercial sources restored the lack of Th17 cells, implying an essential role for a transmissible component of the microbiota. Analysis of the composition of the microbiota of several strains of mice revealed the presence of segmented-filamentous bacteria (SFB) to be necessary and sufficient for the generation of Th17 responses in the small intestines of mice 162. SFB belong to a currently uncultivatable group of spore-forming Gram-positive bacteria related to *Clostridia*, and SFB are resident in the flora of the terminal ileum in many mouse colonies, including those maintained under SPF conditions. Mechanistically, it has been shown that SFB may drive Th17 accumulation in the small intestinal LP via a serum amyloid A protein-dependent effect, resulting in IL-6 and IL-23 production from dendritic cells 162. Other groups have reported that SFB may exert more diverse effects on intestinal T-cell populations, including Th1 and Treg cells 163. Interestingly, in mice expressing human α-defensin, reduced Th17 populations were associated with a reduced intestinal burden of SFB, whereas Th1 cells were unaffected 164.

The molecular and functional attributes of SFB inducing the development of Th17 cells remain unclear. The recent sequencing of the SFB genome may result in important insights into functional aspects of microbial determinants of intestinal T-cell development 165, 166. Whether additional species of bacteria are similarly able to induce intestinal Th17 accumulation awaits further study. Importantly, colonization of germ-free mice with a defined cocktail of innocuous bacteria (excluding SFB) contained in altered-Schaedlers flora was able to promote Th17 accumulation when IL-10 signaling was blocked, demonstrating that Th17 responses can be generated in the absence of SFB when regulatory pathways are defective 167.

The presence of Th17 cells in mice lacking key molecules involved in the sensing of microbes makes it quite unclear how the necessary host–microbiota interactions are mediated. MyD88^−/−^Trif^−/−^ double knockout mice, which lack functional signaling via the Toll-like receptor pathway, generate normal Th17 responses in the steady state 66, 68. Similarly, deficiency of receptor interacting protein 2 (RIP2), an adapter of the nucleotide oligomerization domain (NOD) pathway, does not impair Th17 development 162. Intriguingly, *Tlr9*^−/−^ mice are reported to have altered mucosal Th17/Treg ratios, but this is difficult to reconcile with the reported lack of effect of MyD88 deficiency, through which TLR9 signals 168. Similarly, interaction with microbes via other mechanisms, including sensing of bacteria-derived ATP, has been proposed, but is unlikely to explain the specific effect of SFB as it does not activate this pathway 68. Furthermore, the requirement for specific pathways to drive Th17 cell development in conditions of infection or inflammation may differ. TLR1 has been reported to be required for the generation of protective Th17 responses in oral *Yersinia enterocolitica* infection, via an IL-6- and IL-23-dependent mechanism 169. An important unresolved issue is the antigen specificity of Th17 cells generated in response to microbial colonization. It has been suggested that intestinal Th17 cells arise in the absence of cognate antigen, but whether this is a general phenomenon remains to be determined 170.

## Treg cells

Similar to the observations made in studies of Th17 accumulation during ontogeny 66, intestinal Foxp3^+^ or IL-10^+^ cell numbers increase rapidly in the first month of life in mice, with a rapid accumulation seen at the time of weaning 171. In contrast to observations for Th17 cells, Treg populations are maintained in the small intestines of germ-free animals 66, 171, 172. By contrast, Treg cells within the colon are highly dependent on the presence of the flora, being significantly reduced in germ-free conditions or by antibiotic treatment, and accumulating when germ-free mice are ‘conventionalized’ by feeding fecal pellets from SPF animals. In such studies, analysis of specific components of the flora has implicated a key role for members of the clostridium family, particularly clusters IV and XIVa. Colonization with a defined flora composed of 46 *Clostridium* species was sufficient to induce accumulation of CTLA-4^hi^ IL-10^+^Helios^−^ iTreg cells in the colon 171. Notably, achieving greater levels of colonization resulted in more pronounced Treg activity and protection in DSS and oxazolone-induced colitis. Although species-specific differences in the composition of the microbiota might limit the translational significance of these findings, it is notable that patients with IBD have a lower representation of clostridia within their flora, including reduced clostridium clusters IV and XIVa 173, 174.

No difference in the small intestinal Treg population was seen in either germ-free mice or those colonized with the cocktail of clostridia, suggesting important differences in the biology of regulatory cells exists within discrete compartments of the intestinal tract. Furthermore, as is the situation for Th17 cells the relevant pathways driving host–microbial interaction in this scenario are uncertain, as Treg populations were unaffected by deficiency of MyD88, Rip2, or Card9 171.

Colonization with the human symbiont *Bacteroides fragilis* has been reported to protect mice from *Helicobacter hepaticus*-driven pathology in a variation in the T-cell transfer model, via the induction of IL-10 production from CD4^+^ T cells 175. This protection was dependent on microbial expression of polysaccharide A (PSA). Monocolonization with *B. fragilis* was reported to induce functional IL-10 producing iTregs in a PSA and TLR2-dependent manner 176, 177. Interestingly, such cells did not produce TGF-β1 or the Ebi-3 component of IL-35, but were able to afford protection in the TNBS model of colitis. Notably, the effect of PSA on mucosal T cells, favoring regulatory responses, contrasts with its Th1-promoting effects on cells within secondary lymphoid tissues 178. The intestinal bacteria *Faecalibacterium prausnitzii*, isolated from the flora of patients with CD, has also been shown to promote IL-10 production, suggesting this characteristic may be conserved across multiple species of bacteria 174.

## Retinoic acid

An important role for the vitamin A derivative retinoic acid (RA) in intestinal immune responses has been demonstrated in numerous experimental systems. Such studies highlight multiple mechanisms by which RA might exert its effects.

Mice with impaired RA signaling or those fed diets deficient in vitamin A have reduced populations of activated T cells in the intestinal LP 179. This may reflect a requirement for RA for the upregulation of gut-homing molecules including α_4_β_7_ and CCR9 on T cells. This imprinting is mediated by populations of specialized macrophages and DCs within the LP and GALT, characterized by the expression of CD103 (α_E_-integrin), and capable of metabolizing vitamin A to RA via retinal dehydrogenases (RALDH) 18. Mucosal and MLN CD103^+^ DCs are enriched for basal expression of *Aldh1a2*, encoding RALDH2, whereas CD103^+^ DCs present in other immune compartments express much lower levels of RALDH2. Interestingly, RALDH expression may be linked to the presence of the intestinal flora, with TLR2 activation shown to be a particularly potent inducing signal 180. Furthermore, intestinal epithelial and MLN stromal cells are also capable of metabolizing RA and may support local conditioning of DCs 18, 181. Therefore, multiple specialized cell populations within the intestine may be involved in the regulation of RA metabolism and hence influence downstream effects on T cells.

In addition to effects on T-cell homing, RA may also directly influence the differentiation and development of T-cell subsets in the intestine. Along with TGF-β1 and IL-2, RA acts as a cofactor in the generation of iTregs, also via a CD103^+^ APC-dependent mechanism 18. Vitamin A-deficient mice demonstrate impaired iTreg generation in response to oral antigen feeding in an oral tolerance model 182. However, the effect of vitamin A deficiency on induction of oral tolerance may relate to combined effects on both homing and iTreg development 183. Vitamin A-deficient mice have maintained overall Treg cell populations, and IL-10^+^ Tr1 cells are unaffected, implying the requirement for RA signals is specific to Foxp3^+^ iTreg cells 182, 184. However, RA alone is insufficient to induce upregulation of Foxp3, and RA requires the additional presence of TGF-β1 179. The mechanism underlying this synergy may reflect activity of both RA and TGF-β1 on SMAD3 pathways.

Conversely, RA may inhibit the Th17 axis, via inhibition of IL-6 and IL-23R expression and consequent repression of Th17 development 179. However, it is notable that steady-state intestinal Th17 populations are ablated in vitamin A-deficient mice, associated with reduced levels of the cytokine IL-6. These data therefore suggest that RA may exert paradoxical effects on Th17 cells related to the nature of the exposure and additional factors, such as activation status and prevailing cytokine environment. In support of this concept is the interesting observation that in the presence of high levels of IL-15, a scenario observed in the intestine in human celiac disease, RA acts as an adjuvant to suppress iTreg generation while favoring effector T-cell responses 185. RA acted at the level of the DC, increasing IL-15-driven IL-12 and IL-23 production, and directly on T cells, where it synergized with these downstream cytokines, favoring Th1 and Th17 responses.

## Manipulation of intestinal T cells in the treatment of IBD

In view of the central role of CD4^+^ T cells in intestinal homeostatic or inflammatory responses, manipulation of constituent subsets may be a highly effective therapeutic approach. Intestinal CD4^+^ T cells isolated from the inflamed intestine in IBD demonstrate relative resistance to apoptosis 186. Therefore, approaches to reinstate sensitivity to induction of apoptosis represent an important potential mechanism of action for novel therapeutics.

The efficacy of two major classes of drugs currently used in the treatment of IBD has been linked to induction of apoptosis in CD4^+^ T cells. Anti-TNF monoclonal antibodies induce rapid apoptosis in colonic mucosal T cells *in vivo*, and this has been linked to therapeutic efficacy 187. Furthermore, whereas anti-TNF drugs which do not induce T-cell apoptosis are effective in treating other systemic inflammatory diseases, their lack of benefit in IBD has been suggested to reflect a specific and critical requirement for this mechanism of action for therapeutic efficacy in IBD 188.

Among multiple other roles, IL-6 is proposed to be an important mediator of antiapoptotic pathways in IBD in a STAT3-Bcl-dependent manner 189. Antibody blockade of IL-6R signaling renders T cells susceptible to apoptosis, and treatment with an anti-IL-6R antibody suppressed colitis in the T-cell transfer model 88. Investigation of the efficacy of anti-IL-6 therapies in IBD is limited.

Another major class of drugs currently used in the treatment of IBD, the thiopurines (azathioprine and 6-mercaptopurine), may also exert their effects via induction of CD4^+^ T-cell apoptosis *in vivo* through a mechanism involving the CD28-Rac-1-Vav1 pathway 190.

The complementary approach of preventing accumulation of CD4^+^ T cells in the gut through targeting of molecules involved in the homing of cells to the intestine is currently the subject of intense interest. Trials of anti-α_4_ integrin treatment have demonstrated efficacy in active CD, but their widespread use in IBD is prevented by concerns over their safety profile 191, 192. In particular, a number of patients treated with anti-α_4_ integrin therapy have developed the fatal neurological disease progressive multifocal leukoencephalopathy, associated with JC-polyomavirus reactivation, presumably reflecting the role of α_4_ in T-cell homing to the central nervous system 192. Trials of agents targeting more intestine-specific homing molecules including anti-β7, anti-MadCAM1, and anti-CCR9 are ongoing in human IBD, with promising initial observations 193.

Among anticytokine therapies, anti-TNF remains the most efficacious, although many patients are non-responsive 194. As mentioned earlier, anti-IL-17A was not effective in CD 96, and it may be that stratification of patients with distinct ‘pathotypes’ of disease is required to see beneficial effects in patient subgroups with blockade of particular effector cytokines.

In addition to inhibiting pathological T-cell responses, complementary therapies may be necessary to restore and maintain intestinal homeostasis. These may include strategies to enhance intestinal barrier function and the number and function of intestinal Treg cells. As these effects should be focused on the intestine, manipulation of the microbiota may offer a tool to deliver these goals and evidence from model systems is encouraging 25.

## Conclusions

Recent advances have contributed significantly to our understanding of the biology of CD4^+^ T cells in the intestine. These cells fulfill both homeostatic and pathological functions and display highly context-specific roles. Interestingly, it is increasingly apparent that these subsets are more complex than previously appreciated and are subject to multiple layers of regulation by host and environmental factors, particularly components of the microbiota. At the same time, the paradoxical roles of specific subsets of cells or their mediators in homeostatic or inflammatory settings urges caution in attempts to translate interim findings in model systems to human disease. However, the increasingly sophisticated approaches available, such as cell-specific genetic ablation and animals carrying a defined microbiota, offer the opportunity to continue the pace of recent advances in the future. A better understanding of the origins, regulation, and role of intestinal T cells, both in local disease and in systemic conditions such as arthritis and diabetes, carries significant prospects for the development of future therapeutic interventions.
